# Accuracy of automated 3D cephalometric landmarks by deep learning algorithms: systematic review and meta-analysis

**DOI:** 10.1007/s11547-023-01629-2

**Published:** 2023-04-24

**Authors:** Marco Serafin, Benedetta Baldini, Federico Cabitza, Gianpaolo Carrafiello, Giuseppe Baselli, Massimo Del Fabbro, Chiarella Sforza, Alberto Caprioglio, Gianluca M. Tartaglia

**Affiliations:** 1 Department of Biomedical Sciences for Health, University of Milan, Via Mangiagalli 31, 20133 Milan, Italy; 2 Department of Electronics, Information and Bioengineering, Politecnico Di Milano, Via Ponzio 34/5, 20133 Milan, Italy; 3 Department of Informatics, System and Communication, University of Milano-Bicocca, Viale Sarca 336, 20126 Milan, Italy; 4 Department of Oncology and Hematology-Oncology, University of Milan, Via Sforza 35, 20122 Milan, Italy; 5 Department of Biomedical, Surgical and Dental Sciences, University of Milan, Via della Commenda 10, 20122 Milan, Italy; 6 Fondazione IRCCS Cà Granda, Ospedale Maggiore Policlinico, Via Sforza 35, 20122 Milan, Italy; 7 IRCCS Istituto Ortopedico Galeazzi, Via Belgioioso 173, 20157 Milan, Italy

**Keywords:** Landmark, Three-dimensional imaging, Orthodontics, Maxillofacial, Deep learning

## Abstract

**Objectives:**

The aim of the present systematic review and meta-analysis is to assess the accuracy of automated landmarking using deep learning in comparison with manual tracing for cephalometric analysis of 3D medical images.

**Methods:**

PubMed/Medline, IEEE Xplore, Scopus and ArXiv electronic databases were searched. Selection criteria were: ex vivo and in vivo volumetric data images suitable for 3D landmarking (Problem), a minimum of five automated landmarking performed by deep learning method (Intervention), manual landmarking (Comparison), and mean accuracy, in mm, between manual and automated landmarking (Outcome). QUADAS-2 was adapted for quality analysis. Meta-analysis was performed on studies that reported as outcome mean values and standard deviation of the difference (error) between manual and automated landmarking. Linear regression plots were used to analyze correlations between mean accuracy and year of publication.

**Results:**

The initial electronic screening yielded 252 papers published between 2020 and 2022. A total of 15 studies were included for the qualitative synthesis, whereas 11 studies were used for the meta-analysis. Overall random effect model revealed a mean value of 2.44 mm, with a high heterogeneity (*I*^2^ = 98.13%, *τ*^2^ = 1.018, *p*-value < 0.001); risk of bias was high due to the presence of issues for several domains per study. Meta-regression indicated a significant relation between mean error and year of publication (*p* value = 0.012).

**Conclusion:**

Deep learning algorithms showed an excellent accuracy for automated 3D cephalometric landmarking. In the last two years promising algorithms have been developed and improvements in landmarks annotation accuracy have been done.

## Introduction

Radiographic exams are considered essential for orthodontic and maxillofacial treatments; however, diagnostic values of conventional radiographs and indications for their use are controversial, especially when radiation exposure is related to pediatric patients. To solve the appropriateness of orthodontic radiographs, specific guidelines were recently provided [1].

Cephalometric analysis is a quantitative diagnostic tool that is daily used by orthodontists and maxillofacial surgeons to evaluate skeletal and dentoalveolar relationships, morphometrical characteristics, and growth pattern of their patients [2]. It was first introduced in 1931 and since then it has been evolving until the latest finding in orthodontic radiology and diagnostics [3]. This method has been based on both linear and angular measurements conventionally taken on two-dimensional (2D) radiographs of the skull, producing an individual cephalogram for each patient. Conventional reference points for cephalometry are marked on skeletal structures like the anterior and posterior cranial base or the maxilla and mandible, on teeth like molars and incisors, and on the soft tissue-like structures among nose and chin; distances and angles between pointed landmarks, as well as axes and planes, allow to classify individual patients in accordance with skeletal, dental and profilometric features. The gold standard for performing this procedure is still the manual tracing of these specific points relative to meaningful anatomical structures of skull and neck, visualizing them on lateral, frontal, and axial views of 2D radiographs. The main issues related to accurate identification of the cephalometric points are represented by the time and high level of expertise required, and the risk of intra- and inter-operator variability [4].

Given the crucial role of cephalometric analysis in treatment planning, it's noteworthy that inaccuracies in landmarking can lead to incorrect measurements of distances and angles between reference points [5]. As a result, misidentification of landmarks and consequent errors in measurement can not only result in incorrect diagnoses but also inappropriate treatment planning and suboptimal treatment outcomes, such as over- or under-correction of the malocclusion, changes in facial esthetics, or functional issues.

Since the introduction in dentistry and maxillofacial surgery of cone beam computed tomography (CBCT), the cephalometric analysis can also be performed by three-dimensional (3D) visualization and identification of the landmarks. In 1995 3D analysis started by soft tissue and progressively moved to bone until it became what it is known as 3D cephalometric analysis. However, there is no 3D conventional or validated list of anatomical landmarks, also because 3D data made it possible to identify hidden structures to 2D analysis. Despite that, the main advantage represented by 3D analysis is to avoid the superimposition of bilateral structures and the distortion caused by the representation of a 3D object into a 2D image, resulting in a greater accuracy [6]; furthermore, CBCT technology in orthodontics allows the reduction of the X-rays exposition due to reduction of field of view and through the use of new reference landmarks and planes [7].

Because manual landmarking is a time-consuming task, automated detection of landmarks could be certainly helpful, as it facilitates access to the cephalometric analysis even it represents a challenge for the biomedical engineering field. Artificial intelligence (AI) applications are becoming increasingly common in dentistry especially on image analysis [8], and it has been an active research field over the last years [9]. Orthodontics, as well, is one of the dentistry branches most involved in this field by means of different AI algorithms for diagnosis and treatment planning [10]. Advances in medical imaging technologies and methods allow AI to be used in orthodontics to shorten the planning time of treatment, including tooth segmentation on CBCT images or digital casts, classification of defects on X-rays images, and automatic search of landmarks for cephalometric analysis [11].

The main issues in developing AI algorithms for 3D cephalometry are the increased number of parameters, the need for high-performance computing, and the greater computational complexity that increase subsequently of the clinical requests for more accurate and faster analyses. Recently, two systematic reviews on the accuracy of automated cephalometric points identification have been published: Dot et al. [12] compared different automated methods for analyzing 3D images, while Schwendicke et al. [13] evaluated deep learning (DL) methods for analyzing 2D and 3D radiographs; it was by both of them reported that DL-based methods yielded promising results compared to older techniques like knowledge-based, atlas-based or learning-based methods. The image analysis approach is quite similar to all automated methods: determination of the region of interest where the landmark is potentially located, determination of the landmark positioning on the 3D model surface, finally confirming and adjusting on sectional or cutaway views to reduce the mean error of the landmarking; nevertheless, the difficulty in the computational identification depends on the fact that cephalometric landmarks have different anatomical characteristics, being located on surfaces, in the space or within the bone cavity. Most of the analyzed studies included in these reviews reported a great accuracy between manual and automated landmarking, often under 2 mm threshold of clinically acceptability [14–16]; reviewing previous scientific literature about this field reported an accuracy depending on the algorithm used for the automation in landmarking: knowledge-based method shown an accuracy ranging from 1.88–2.51 mm [17–19], registration-based method a mean error between 1.99–3.4 mm [20, 21], machine learning (ML) ranged between 1.44–3.92 mm [22, 23], whereas the best results were offered by DL method with error ranging between 0.49–1.80 mm [14, 15, 24]. Probably, the variability in the accuracy between studies may depend not only on the type of artificial neural network (ANN) but also on the amount of CBCT data, the number and type of landmarks analyzed. Considering the excellent performances of DL studies published before 2020, we focused our research on studies that used DL algorithm for this purpose, in order to better analyze performances in a larger number of studies. Unfortunately, the aforementioned systematic reviews have not yet been updated since 2021.

Despite the number of studies involving DL for automated landmarking is increasing day by day, the accuracy of their results remains unclear; a previous systematic review analyzed both ML and DL methods on 3D images [12], whereas another one focused on DL algorithms applied on 2D or 3D cephalometric analysis [13]. To the best of our knowledge, new studies have been carried out on the last two years increasing the data available for accuracy comparison between them. Therefore, the aim of the present systematic review and meta-analysis is to assess the accuracy of automated landmarking using DL in comparison with manual tracing for cephalometric analysis of 3D medical images. The aim of the present research was to investigate the accuracy of DL-based algorithm for automatic identification of cephalometric landmarks on 3D radiographs. Implications related to the present topic may help radiologists in identifying the maximum mean error tolerated by future AI systems for automated landmarking, as well as to show the possibility to erase intra- and inter-observer errors that frequently affect manual landmarking and subsequent cephalometric analysis. The combination of greater precision and the exclusion of intra- and inter-operator errors can lead to a more accurate diagnostic process that ends with better quality in treatment, esthetics and functionality.

## Materials and methods

### Protocol and registration

The present systematic review was registered to the PROSPERO database (registration number CRD42022315312). The reporting of this study is in accordance with PRISMA statement [25] and followed the guidelines in the Cochrane Handbook for Systematic Reviews of Interventions [26].

### Eligibility criteria

The selection criteria were structured according to the PICO (Problem, Intervention, Comparison, Outcome) format: ex vivo and in vivo volumetric data images (CBCTs or CTs) suitable for 3D landmarking of osseous cranial reference points for cephalometric purpose (*P*), a minimum of five automated landmarking performed by DL method (*I)*, manual landmarking (ground truth) performed once or more times by one or more trained operators or annotated images from previous studies (*C*), and intergroup mean accuracy, expressed in mm, between manual and automated landmarking (*O*). All studies that did not include the main outcome, reporting partial data, or non-English written were excluded. Thus, the selection criteria applied were: accuracy studies based on DL algorithm, using 3D images (CT or CBCT) with a minimum of 5 landmarks to be detected, reporting the outcome as mean error between automatic and manually performed landmarking, published between 2020 and 2022.

### Information sources and study selection and Data Collection

In order to get only the most recent evidence, articles published from January 1, 2020, to December 31, 2022, were searched, and those already cited in the previous reviews were not included. The following electronic databases were screened: PubMed/Medline, Web of Science, IEEE Xplore, Scopus and ArXiv. The combination of different Boolean operator AND/OR and MeSH/non-MeSH terms was used to select appropriate studies: [deep learning] AND [landmarking], [3D cephalometry] AND [deep learning], [CBCT] OR [CT] AND [automated cephalometry], [deep learning] AND [tomography], [automatic] AND [3D cephalometric analysis]. The last electronic search was performed on January 27th, 2023. Additional studies were selected by searching the reference lists of all included articles, and all related papers were also screened through the PubMed database. EndNote software (EndNote X9; Clarivate™, Philadelphia, PA) was used to collect references and remove duplicates. The study selection was independently carried out by two reviewers (MS and BB) and evaluated through Cohen’s Kappa coefficient; any disagreement was solved by a third expert reviewer (CS). The same two reviewers extracted study characteristics, such as authors, year of publication, algorithm architecture, type and number of images included in the dataset, dataset partition (training and test), number of landmarks aimed to detect, accuracy metrics defined as total maximum, minimum and mean difference between manual and automated landmarking.

### Risk of Bias Assessment and Level of Evidence

Risk of bias and applicability concerns were assessed by QUADAS-2 tool [27], whereas GRADE criteria were used to assess the overall quality of the evidence. The two reviewers evaluated independently all the included studies. Any disagreement was solved by discussion. The risk of bias was evaluated for Data Selection, Index Test, Reference Standard and Flow and Timing, while applicability concerns regarded Patient Selection, Index Test and Reference Standard. These domains were judged as low risk, unclear risk, and high risk, while the overall quality of the evidence was categorized as high, moderate, low, and very low.

### Meta-analysis

Meta-analysis was performed on the studies that reported as outcome mean and standard deviation values of error between manual and automated landmarking. Meta-analysis was performed in Prometa3 (ProMeta 3 – IDoStatistics), while the graphs were realized in Excel (Microsoft Corporation. (2018). Microsoft Excel. Retrieved from https://office.microsoft.com/excel). It was conducted using random-effects model, that takes two sources of variance into account: the within-study variance and the between-studies variance. For each study, the considered parameters were mean difference, standard deviation and sample size (number of considered landmarks). Graphic display of the estimated mean error between studies in conjunction with the 95% confidence interval (CI) was obtained. Heterogeneity was assessed by I^2^ and τ^2^ statistics, using random-effect models [28]. Linear regression plots were also used to analyze any correlation between the mean accuracy and the year of publication. *p* values < 0.05 were considered statistically significant.

## Results

### Study selection and qualitative analysis

**Fig. 1:**
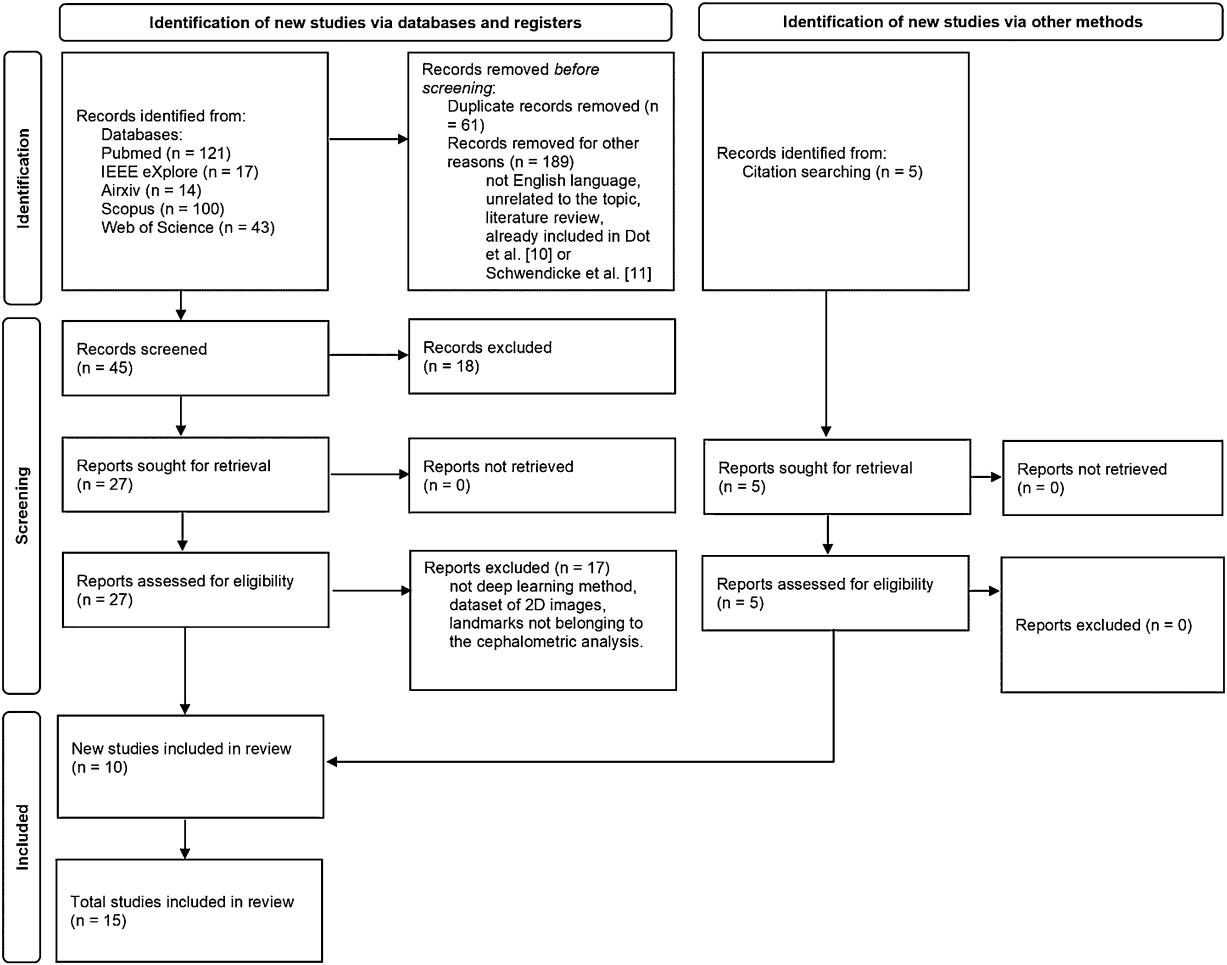
Prisma flowchart for the papers’ selection process

Figure 1 reports the PRISMA flowchart of the study selection process. A total of 295 studies (121 PubMed, 17 IEEE Explore, 14 ArXiv, 100 Scopus, 43 Web of Science) were initially selected at the first screening; 280 studies suitable for eligibility were subsequently excluded in accordance with exclusion criteria. After a final step selection, a total of 15 studies were included for the qualitative synthesis, whereas 11 studies were used for the meta-analysis. A final Cohen’s K coefficient of 0.96 was achieved as a result of the double-blind search.

All articles were retrospective studies published during years 2020–2022 and employed DL models. All of the studies used convolutional neural network (CNN) architectures modified or combined with other architectures. Almost all studies used as dataset images paired with the correspondent set of landmarks. Six studies used an image dataset of CT, two studies CBCT and four studies used both. One study used as dataset both annotated CBCT images (labeled images) and not-annotated ones (unlabeled) [29] and three studies [30–32] used a dataset composed by labeled CT images and a landmark dataset of the 3D positions of landmarks from CT. One study reported the mean error in pixels dimension [33] instead of mm, and for this review, it was converted in mm using the pixel-to-mm conversion rate reported in the article. Studies detected a mean (± standard deviation) of 47(± 35) landmarks, with a 5–105 range. On average, the size of the image dataset was 88 (± 53), 24–198 range. Description of the sample characteristics (e.g., age, gender, craniofacial characteristics) was not provided by all the articles. Only eight studies [29, 34–40] indicated the execution time of the identification system, and four of them [34, 36, 38, 39] also reported the duration of the training phase.

Twelve studies provided information about the computer system used. The reference standard used was manual landmarking for all of the studies, it was established by two experts in five studies, by one expert in four studies and in one study part of the dataset was annotated by one expert and the rest by three experts. In five studies the number of experts who annotated the images was unclear. Only one study [40] indicated the inter-rater agreement. For eleven studies the reported outcome was the mean error and the standard deviation between manual and automatic landmarking and they were included in the meta-analysis [29, 30, 33–41]. Four studies didn’t report the standard deviation [31, 32, 42, 43]; thus, they were excluded. Detailed information about studies’ characteristics can be found in Table 1.

**Table 1:** Characteristics and details of the studies included in the systematic review. Abbreviations: SD, Standard Deviation; CNN, Convolutional Neural Network; VAE, Variational AutoEncoder; CT, Computed Tomography; PCA, Principal Component Analysis; DTNet, Multi-task Dynamic Transformer Network; CBCT, Cone Beam Computed Tomography; DRL, Deep Reinforcement Learning; FCN, Fully Convolutional Network; R-CNN, Region-based CNN; MS-UNet, Multi-Scale UNet; SCN, SpatialConfiguration-Net; SA-LSTM, Structure-Aware Long Short-Term Memory network; LDL, Local Dependency Learning

Author(s) and year	Algorithm architecture	Programming Language	Dataset Sample size	Training dataset	Test dataset	N. landmarks	Total Mean difference ± SD [mm]	Maximum mean difference ± SD [mm]	Minimum mean difference ± SD [mm]
Yun et al. [28]	Shadowed 2D-image-based CNN + image-based CNN + patch-based CNN + VAE	Unknown	26 CT	22	4	93	3.63 ± 1.41	7.47	1.41
Yun et al. [29]	Shadowed 2D-image-based CNN + VAE + 3D patch-based CNN + 2D CNN + VAE	Python (Pytorch library)	24 CT	15	9	90	2.91	Unknown	Unknown
Ma et al. [32]	PCA + patch-based CNN	MATLAB for PCA, Python 3.6 (Tensorflow 1.9.0 and Keras 2.1.4 libraries)	66 CT	50 (training) + 8 (validation)	8	13	5.785 ± 0.980	Unknown	Unknown
Lian et al. [33]	DTNet	Python (Pytorch library)	140 (77 CBCT + 63 CT)	120	20	64	2.52 ± 0.31	Unknown	Unknown
Kang et al. [34]	Single- or Multi-stage DRL	Unknown	28 CT	20	8	16	1.96 ± 0.78	2.79 ± 1.14	1.03 ± 0.36
Palazzo et al. [40]	Deep Multi-stage CNN	Python (Pytorch 0.3 library)	19 CT (airways) 50 CT (mandibles)	Unknown, Unknown	Unknown, Unknown	5, 9	0.85, 0.78	1.06, 1.08	0.57, 0.47
Nishimoto et al. [31]	Modified Resnet-3D-50	Python 3.7 (Keras 2.3.1 library)	120 CT	90	30	16	2.81 ± 1.63	Unknown	Unknown
Chen et al. [27]	Region Attention loss CNN + shape adjustment algorithm	Python (Pytorch library)	93 CBCT	62 (31 labeled + 31 unlabeled)	31	33	2.49 ± 1.56	Unknown	Unknown
Zhang et al. [41]	2FCNs	Unknown	107 CT	Unknown	Unknown	15	1.10	Unknown	Unknown
Liu et al. [35]	SkullEngine Network	Python 3.7 (Pytorch 1.7 library)	170 (92 CBCT + 78 CT)	119	17 (validation) + 34 (test)	66	3.03 ± 1.96	Unknown	Unknown
Chen et al. [39]	3D faster R-CNN + MS-UNet	Unknown	80 CBCT	Unknown	Unknown	18	0.79 ± 0.62	1.15	0.75
Dot et al. [36]	SCN	Unknown	198 CT	128 (training) + 32 (validation)	38	33	1.0 ± 1.3	2.7 ± 2.0	0.4 ± 0.2
Chen et al. [37]	SA-LSTM	Python (Pytorch library)	89 CBCT	59	30	17	1.64 ± 1.13	2.28 ± 1.54	0.89 ± 0.52
Lang et al. [38]	3D Mask R-CNN and LDL	Python (Tensorflow library)	50 CBCT	45	5	105	1.38 ± 0.95	1.86 ± 1.04	1.25 ± 0.44
Yun et al. [30]	VAE, 3D CNN	Python (Pytorch library)	24 CT + 229 Landmarks 3D positions	244 (229 landmarks data and 15 CT)	9	90	2.88	Unknown	Unknown

### Quality assessment

Risk of bias and applicability concerns were performed according to QUADAS-2 tool and resumed in Table 2. High risk of bias was found regarding the data selection (*n* = 10), low for reference standard (*n* = 4), index test (*n* = 0) and Flow and Timing (*n* = 0); high risk was also found regarding the applicability concerns in most of the studies toward data selection (*n* = 10), references test (*n* = 4), while low risk for index test (*n* = 0). The high risk of bias for data selection was due to lack of description of patient selection and imaging parameters. As far as the reference standard is concerned, not all articles report how the manual annotation was conducted. Applicability issues for patient selection were due to the fact that the articles did not explain the patient inclusion and exclusion criteria. All included studies were judged to have a critical risk of bias, as issues existed for at least three domains per study. According to GRADE criteria, the overall quality of the evidence was considered low due to the presence of severe risk of bias in the sample selection in the individual studies and discrepancies in the landmark’s selection and definition.

**Table 2:** QUADAS-2 analysis for all the studies included in the systematic review

Author(s) and year	Risk of Bias				Applicability Concerns			Overall Risk of bias
	Data selection	Index test	Reference standard	Flow and timing	Patient selection	Index test	Reference standard	
Yun et al. [28]	High	Low	Unclear	Low	High	Low	Unclear	High
Yun et al. 2020b [29]	High	Low	Unclear	Low	High	Low	Unclear	High
Ma et al. [32]	Unclear	Unclear	High	Low	Unclear	Unclear	High	High
Lian et al. [33]	Unclear	Low	Low	Low	Unclear	Low	Low	Unclear
Kang et al. [34]	Unclear	Unclear	Low	Low	Unclear	Unclear	Low	High
Palazzo et al. [40]	High	Low	High	Low	High	Low	High	High
Nishimoto et al. [31]	High	Unclear	High	Unclear	High	Unclear	High	High
Chen et al. [27]	High	Low	Low	Low	High	Low	Low	Unclear
Zhang et al. [41]	High	Low	Unclear	Low	High	Low	Unclear	High
Liu et al. [35]	High	Low	Unclear	Low	High	Low	Unclear	High
Chen et al. [39]	High	Low	Unclear	Low	High	Low	Unclear	High
Dot et al. [36]	Unclear	Low	Low	Low	Unclear	Low	Low	Unclear
Chen et al. [37]	High	Low	High	Low	High	Low	High	High
Lang et al. [38]	Unclear	Low	Low	Low	Unclear	Low	Low	Unclear
Yun et al. [30]	High	Low	Unclear	Low	High	Low	Unclear	High

### Meta-analysis

**Fig. 2:**
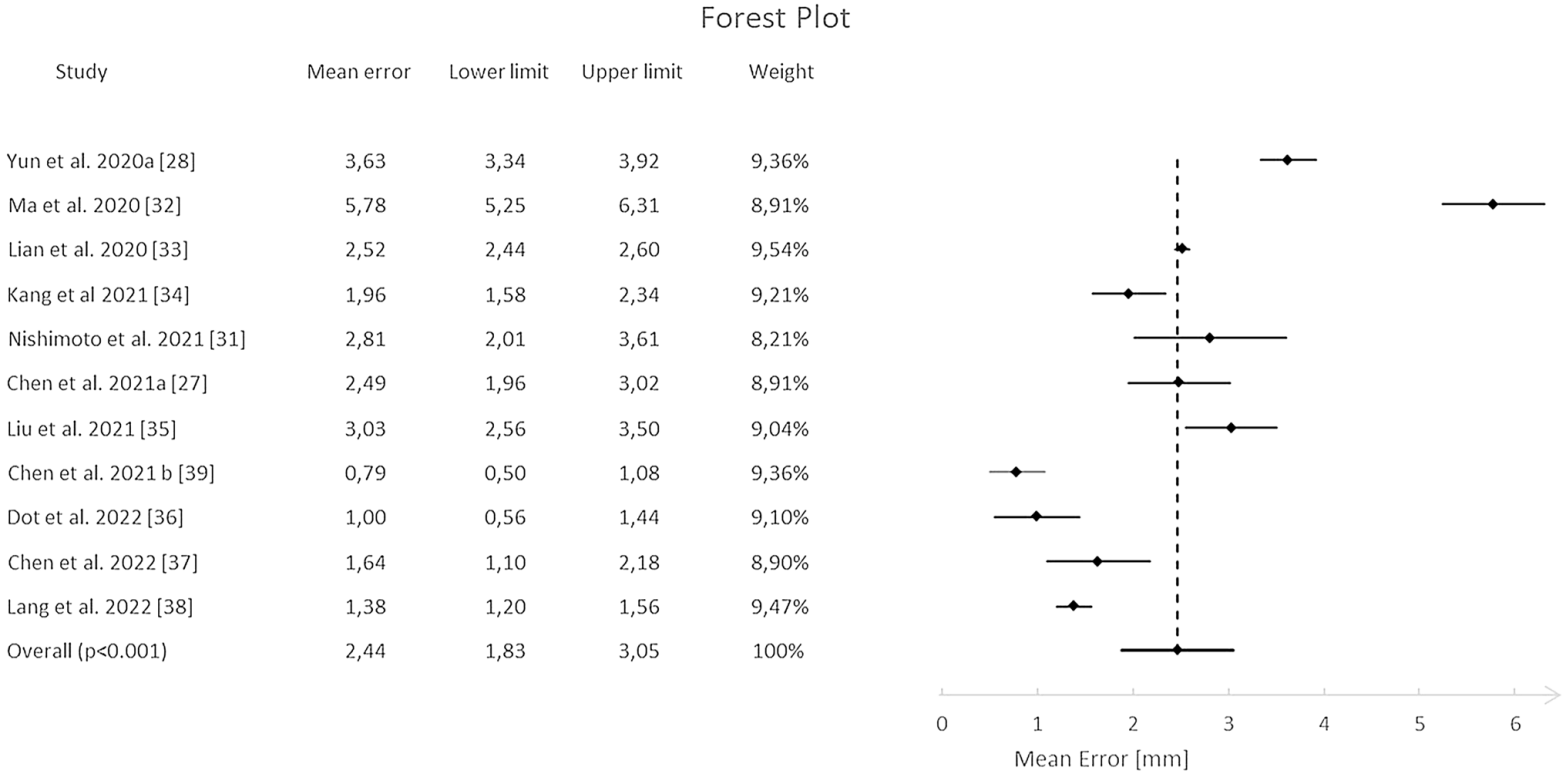
Forest plot reporting the mean error [mm] between manual and automatic landmarking for studies included in the meta-analysis, including relative weights. Indicators represent the mean error for each study and horizontal lines the 95% confidence interval

**Fig. 3:**
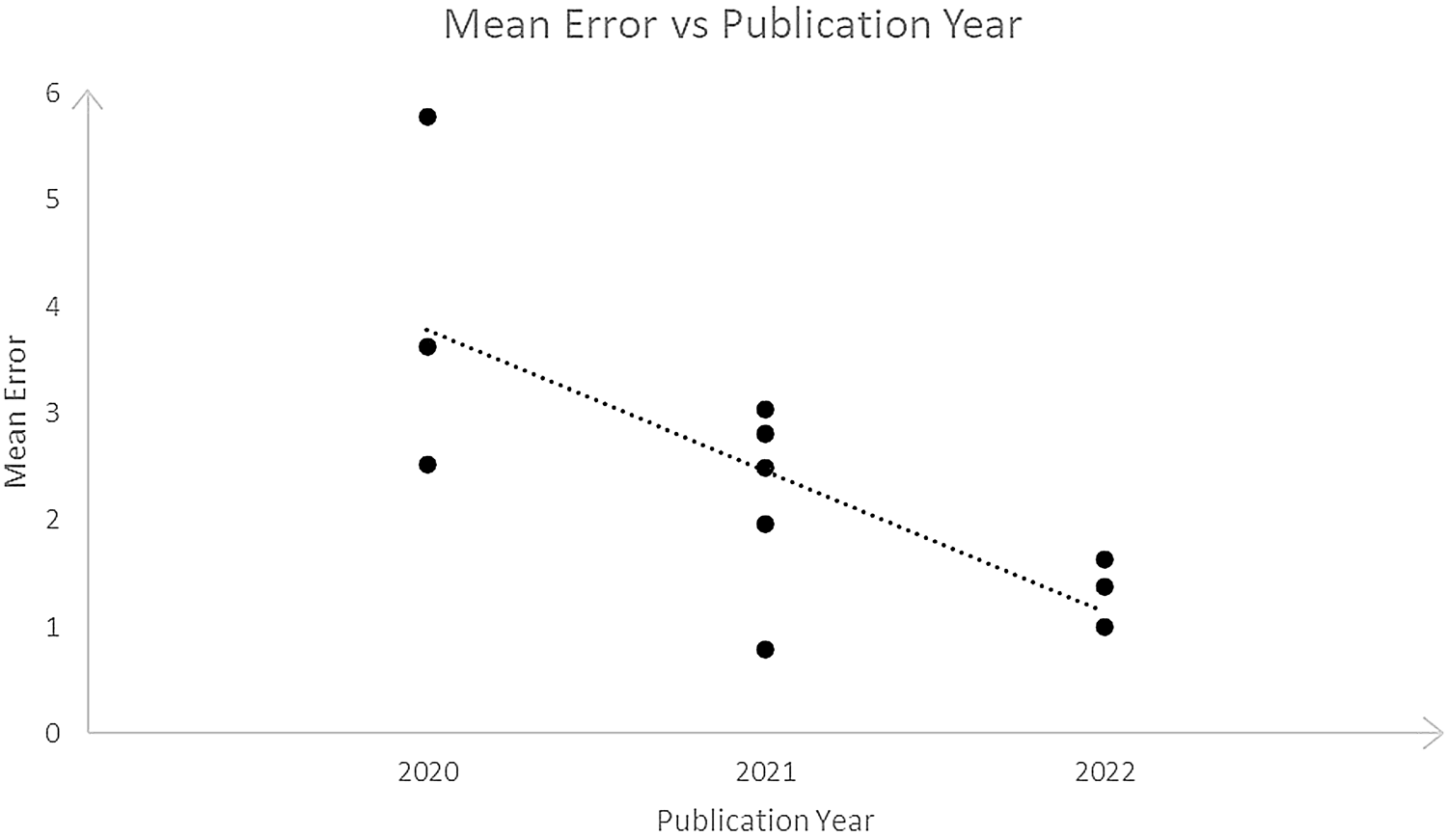
Scatterplot between Mean Error [mm] and Publication Year

A meta-analysis was conducted on eleven studies that presented mean and standard deviation (SD) values of the differences between the automated and manual landmarking. As shown in Fig. 2, the random effect model revealed a mean value (95% CI) of 2.44 (1.83–3.05) mm. Five studies reached a mean value significantly better than the overall effect, whereas three of those have shown a mean value significantly higher. Heterogeneity calculation reported a *I*^2^ = 98.13%, *τ*^2^ = 1.018, *p* value < 0.001. Meta-regression indicated a significant association (*p *value = 0.012) between the mean error and the year of publication, as shown in Fig. 3.

## Discussion

The present systematic review and meta-analysis showed that automated landmarking on 3D radiological images is a promising research field in maxillofacial and orthodontic area for diagnostic purposes. Orthodontic as well as maxillofacial diagnostics are based on clinical evaluation combined with substantial support from radiological imaging techniques. Traditionally, this analysis has been done on 2D images (cephalometries) bringing with it numerous problems in image reconstruction and measurement accuracy, due to the superimposition and distortion of three-dimensional structures projected on a two-dimensional image. Recently, thanks to the introduction of systems for volumetric rendering and the management of large datasets, the interest has been moved toward cephalometric analysis on 3D images, CT or CBCT [44]. However, accurate identification of reference points from X-ray images is used to calculate angular and linear measurements, essential to provide quantitative evaluation of craniofacial structures [45].

In recent years, numerous studies have shown the greater accuracy of 3D cephalometric analysis compared to 2D [46] and the greater efficiency of DL algorithms compared to traditional machine learning methods in the field of bioimages [47], and thus, the trend is to develop DL-based algorithms for automatic identification of points on 3D images. In this context, studies evaluated in this systematic review and meta-analysis showed the update done in the last two years on the automatic identification of craniofacial landmarks on 3D radiographs. Although there is no standard threshold for localization error, the value resulting from the present meta-analysis can be considered a promising result.

To interpret this result, we must consider different sources of bias:

- The overall localization error reported in each paper refers to different type and number (range 5–105) of annotated landmarks. As said before, there is no standard threshold for localization error in 3D cephalometric analysis and, in addition, required accuracy can vary depending on landmark positioning and type, anatomical or geometrical. Moreover, the quality of landmark location and their precise placement is crucial on the reliability of 3D linear and angular measurements [48]; if a landmark is to be used to evaluate a certain dimension, then it should demonstrate a good consistency and precision[49].
- The measurement error can be affected by different types of inaccuracies, thus modifying the clinical significance of the diagnosis and the outcome analysis [46]. Landmarks reproducibility regarding intra- and inter-observer error was previously analyzed for 3D cephalometry [50]. In manual landmarking, it was observed that landmarks’ reliability, reproducibility, accuracy, and precision in the 3D space are affected by the operator, and that 3D references are more reliable than 2D ones. Several features can affect reliability and accuracy, among them the complexity of the model surface, the presence of surrounding structures and landmark type.
- Although in all selected studies the gold standard is manual identification, this is susceptible to human error that is not quantified in the literature. However, from a clinical point of view, the repeatability and reproducibility of manual placements of landmarks with 3D images are acceptable for the majority of anatomical reference points [48].
- Furthermore, since 2D cephalometry is still the gold standard, there is no defined set of points for 3D analysis. In fact, included studies considered different landmarks, so it isn’t possible to make a precise comparison in landmark annotation performance between different used algorithms.
- The performance of DL models crucially depends on the quality of the input dataset, both in terms of quality and appropriateness. The studies included in this systematic review considered different types of datasets: twelve studies used annotated images (pair of images and sets of reference points referred to it) in a supervised learning approach; among these, six used CTs, two CBCTs and the remaining both imaging types. Four studies used a semi-supervised approach with a dataset composed of both paired data (annotated images) and unpaired data. Three of these studies used as unpaired data files containing the 3D positions of the landmarks set, while the other used not annotated images. Moreover, there is high variability in dataset sizes, ranging from 24 to 198 items.

Thus, a challenge for the data scientists and DL developers is to train models that can take these sources of bias into account and to prove their robustness and the generalizability on large multicentric dataset. From a clinical point of view, it is important to define standardized study design and a set of landmarks to be used for 3D cephalometric analysis, to reduce bias in comparison between the different models.

In light of these considerations, the high value of *τ*^2^ (*τ*^2^ = 1.018) obtained from the meta-analysis can be interpreted. *τ*^2^ is used to refer to the amount of among-study heterogeneity in a set of studies being analyzed. A high value of *τ*^2^ means that the results of the studies are quite different from one another and that the abovementioned factors are influencing the results and need to be considered. Therefore, each study result reported in this review must be interpreted also considering the characteristics of the investigation. This systematic review with meta-analysis provides a useful insight into the current situation in the field of 3D automatic identification and underlines the need to define standardized protocols.

Another interesting aspect that can be observed from the forest plot in Fig. 2 is that five out of eleven studies are on the left side of the graph, i.e., their effect size is lower than the overall effect size. Of these, four studies are the most recently published ones. As evidenced by the presence of numerous articles over the past two years, interest in this type of analysis is rapidly increasing, with great improvements in the identification accuracy. The graph in Fig. 3 shows the error trend during the last 2 years: From an average error of 3.71 mm in 2020 and 2021, it reached 1.84 mm, to arrive in 2022 to an error of 1.34 mm. Considering that, from a clinical point of view, the threshold for the manual reference point is fixed at 2 mm in 2D cephalometry [51], these models can be considered a huge support for the clinician in reducing operator-dependent error. Furthermore, the main advantage will be the reduction of the operating time. In fact, an expert clinician takes 10/15 min for the 3D cephalometric annotation, while using DL-based automatic models the time would be significantly reduced to about 1 min or less. This would allow the dentist/orthodontist to speed up time consuming and cumbersome procedures and devote time to patient care and well-being.

One aspect of the current review is the exclusion of diagnostic tools like conventional 2D radiographs, as previously purposed by a recent review [52], in favor of 3D imaging methods. CBCT and CT technologies can surely solve main problems related to bidimensional image analysis: loss of third dimension that results in anatomical structures overlapping, image distortion and non-real measurements quantifications [53, 54].

The main limitations of the present systematic review and meta-analysis are related to the studies included for qualitative and quantitative assessment. The number of studies included in the review may be limited due to the relatively recent emergence of DL and automated landmarking in 3D cephalometry. This can potentially limit the generalizability of the findings and limit the ability to draw definitive conclusions. Furthermore, the included studies have different designs, especially regarding number and type of landmarks, and DL algorithms, which can introduce variability in the results. This can make it difficult to compare and synthesize the findings across studies to be also reliable into clinical reality. Statistical inferences for each specific landmark couldn’t be investigated since the number and type of examined landmarks varied across studies, and not all the studies reported localization errors related to each specific landmark.

## Conclusion

Orthodontic diagnosis is a process that takes a long time, as it includes the analysis and review of radiographic recordings and photographs, model analyses, and patient examination. Hence, these diagnostic methods have to be automated in order to enhance consistency, accuracy, and speed.

DL algorithms have shown a greater accuracy for automated 3D cephalometric landmarking with respect to other ML algorithms. In the last two years, promising DL models have been developed and improvements in landmarks annotation accuracy have been achieved. Despite all the discussed sources of bias, the result of the present meta-analysis is promising from both a clinical and a technological point of view: Clinicians can benefit from an automatic support in 3D cephalometric analysis in terms of excellent intra-operator accuracy and lower time. The development of efficient automatic DL-networks will play an important role in the emerging field of digital dentistry.

One area of future expansion is the improvement of the accuracy and efficiency of automated landmarking through the development of more sophisticated deep learning algorithms. By training models on larger and more diverse datasets, these algorithms could potentially improve the reliability and reproducibility of cephalometric analyses. Additionally, DL-based methods could enable the identification of new anatomical landmarks, which could provide additional information for clinical decision-making.
